# The voice of few, the opinions of many: evidence of social biases in Twitter COVID-19 fake news sharing

**DOI:** 10.1098/rsos.220716

**Published:** 2022-10-26

**Authors:** Piergiorgio Castioni, Giulia Andrighetto, Riccardo Gallotti, Eugenia Polizzi, Manlio De Domenico

**Affiliations:** ^1^ Istituto di Scienze e Tecnologie della Cognizione, Via Palestro 32, Roma, Lazio 00185, Italy; ^2^ Departament d’Enginyeria Informàtica i Matemàtiques, Universitat Rovira i Virgili, Tarragona 43007, Spain; ^3^ CoMuNe Lab, Fondazione Bruno Kessler, Via Sommarive 18, Povo, Trento 38123, Italy; ^4^ Department of Physics and Astronomy ‘Galileo Galilei’, University of Padova, Padova, Italy

**Keywords:** computational social science, social networks, data analysis, fake news, social psychology

## Abstract

Online platforms play a relevant role in the creation and diffusion of false or misleading news. Concerningly, the COVID-19 pandemic is shaping a communication network which reflects the emergence of collective attention towards a topic that rapidly gained universal interest. Here, we characterize the dynamics of this network on Twitter, analysing how unreliable content distributes among its users. We find that a minority of accounts is responsible for the majority of the misinformation circulating online, and identify two categories of users: a few active ones, playing the role of ‘creators’, and a majority playing the role of ‘consumers’. The relative proportion of these groups (approx. 14% creators—86% consumers) appears stable over time: consumers are mostly exposed to the opinions of a vocal minority of creators (which are the origin of 82% of fake content in our data), that could be mistakenly understood as representative of the majority of users. The corresponding pressure from a perceived majority is identified as a potential driver of the ongoing COVID-19 infodemic.

## 1. Introduction

The spread of COVID-19, a respiratory disease responsible for the emerging pandemic observed in early 2020 to date [1–3], has led to an increase in misinformation and disinformation about a broad range of topics, from health to technology [4]. However, due to the unprecedented global health crisis we are facing, the rise of fake news has become a global concern, with the potential to affect public health policy and public order [5,6]. The World Health Organization has recognized this phenomenon and has referred to it as ‘infodemic’ [7], a massive volume of news and narratives not necessarily reliable, including a variety of false rumours and unreliable news appearing during a disease outbreak [8,9], identifying in artificial intelligence an invaluable tool to support the global response against it [10]. In fact, coupling misinformation spreading with an ongoing pandemic might be particularly dangerous for public health [11,12], as it erodes trust in institutions [13] and can lead people to turn to ineffective—and potentially harmful—remedies, as well as to engage in risky behaviour for them and for the collectivity (e.g. refusing vaccines or not adopting non-pharmaceutical interventions, such as wearing masks and physical distancing) [14] that substantially increases epidemic spread [8,15,16].

Over the past years, social media platforms have become one of the preferred arenas for public debates. Notwithstanding, they also represent some of the most vulnerable hotspots for infodemics to occur [17–19]. For instance, on social media platforms, false information is typically shared by more users, and travels far more rapidly than reliable information [20]. This condition is further exacerbated by the presence of social bots [21,22]—i.e. automated accounts impersonating humans—that act as magnifiers of noise, conflicts and (mis)information spread [23–29]. Fed by users’ preferences and attitudes, social media algorithms increase the selectivity of the content to which users are exposed, further limiting content verifiability [30]. All this has been shown to provide a fertile ground for the emergence of echo chambers and epistemic bubbles [31], well-formed and highly segregated communities where unsubstantiated rumours can gain increased exposure and become highly resistant to correction [32].

In this paper, we propose a novel view where social norms are suggested as mechanisms promoting the share of misinformation in online settings. Social psychology has long shown the power of social norms—the informal rules that regulate our social life prescribing what we ought or ought not to do—in guiding behaviour, providing extensive evidence about how expectations and actions of people are affected by what they perceive the majority of their peers thinks or does [33–36]. However, this perception of the majority does not need to be necessarily correct to influence people’ thinking and behaviour. Social norms may suffer from biases and misperceptions [37], due to false consensus [38], or pluralistic ignorance [39]. Individuals may, for example, wrongly estimate the opinions and behaviours of others and develop a biased idea of what the majority thinks or does. In these situations misperceived social norms may emerge and still guide people's behaviour. Previous research in social psychology has provided extensive evidence for the role of such misperceived social norms in supporting the persistence of unpopular attitudes and behaviour in several offline, including heavy drinking [40], racial segregation [41], casual sex [42], bullying [43], adolescent delinquency [44] and stigmatization at work [45], and online domains, e.g. driving polarization in online debates [46]. So far, investigation has rarely accounted for social norms to explain the share of misinformation in online settings. Yet, we believe that there are sound reasons for doing so. For example, a misperceived social norm may arise if the voice of ‘few’ users spreading unreliable content is (wrongly) perceived as being representative of the opinions of ‘many’. Once emerged, this norm influences users’ behaviour and increases the likelihood that they will spread questionable content. In this paper, we examine whether there are features of the online communication network—e.g. those based on content sharing—that could act as a structural basis for the development for such an ‘illusion of the majority’ within online communities engaged in the spreading of unreliable content. We suggest here that a mismatch—e.g. both in terms of size and in terms of segmentation—between users responsible for producing unreliable content and those that mainly share it—could create the conditions for the emergence of an illusion of the majority. Specifically, if the former are less than the latter, and the latter are mostly exposed to the former, this may generate a basis on which misperception about majority’s opinion can naturally arise. While exploratory, this work aims at providing support to the idea that such an illusion of the majority can lead to the emergence of social norms shaping misinformation spread, unlocking future opportunities to experimentally test the causal role of social norms in driving (but also potentially curbing) the spread of misinformation.

In this work, we provide a quantitative analysis of empirical human activities gathered from Twitter, a popular micro blogging platform, on unreliable content—such as fake news and conspiracy theories—in the context of COVID-19 and explore which features could support the emergence of an erroneous social norm supporting the sharing of unreliable content in online settings. For this purpose, we collect and analyse 7.7 million retweets belonging to 1.6 million users, and we introduce a criterion for characterizing the users responsible for spreading fake news by means of two groups that we name ‘creators’ and ‘consumers’.

Our choice is supported by empirical evidence provided in the first section of this work. In line with [47], we first show that the size of the two groups differs significantly: the creators are almost 15 000 while the consumers are 93 000, amounting to the 14% and 86% of the fake news spreaders population, respectively. This difference in size is also confirmed by the existing literature on the subject [47–51]. In the second section, we prove that such a definition is solid and suitable even when the underlying socio-technical system is analysed from a dynamic perspective, showing that users tend to mostly remain in the same group and that inter-group switches are mostly temporary. Finally, we analyse the causality relation between the overall volume of fake news and the size of the groups of creators and consumers, finding the latter to be, in fact, a good control parameter to describe the behaviour of spread dynamics of unreliable content.

## 2. Results

### 2.1. Characterizing the separation between creators and consumers

We analysed the interaction networks involving users posting messages related to COVID-19 on a period of time spanning from 22 January to 22 May 2020. We focused our attention on the USA, which is a geographical area particularly active on Twitter, where we have passively collected 7.7 million retweets that allowed us to reconstruct the communication flow network between 1.6 million user accounts. In the electronic supplementary material, we also used data coming from the UK and Italy. The datasets come from the COVID-19 Infodemic Observatory [52] and have been collected through the Twitter Filter API. All the tweets containing a URL were classified using information coming from publicly available sources [16] (for more details see the Methods section).

**Figure 1.**
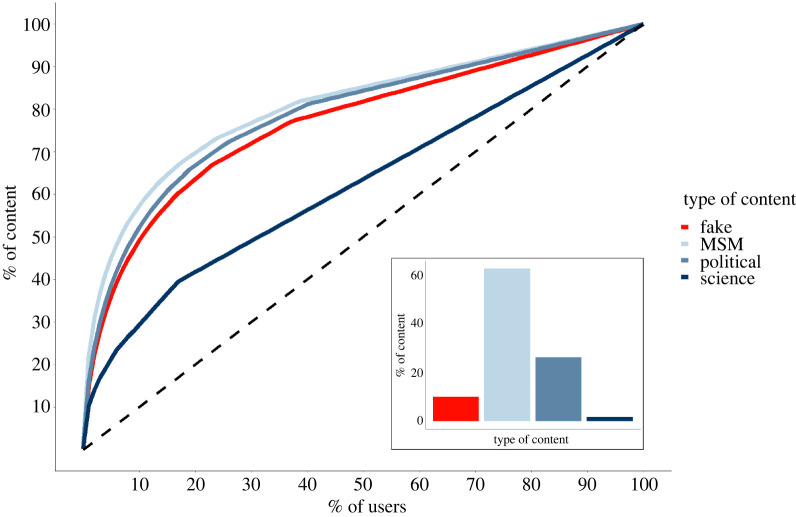
Characterizing the share of content with respect to the share of users who produce them. The *x*-axis indicates the fraction of users ordered from most active to least active, while the *y*-axis displays the share of the overall content (tweets and retweets) that those users are responsible for. The content is divided into four types: fake, mainstream media (or MSM), political and science (see Methods). Different content types are encoded by distinct colours: note the red one, corresponding to content identified as fake news. Black dashed lines correspond to the distribution one would observe if all users were responsible for the same fraction of total content, to highlight the highly heterogeneous activity of content production from different users, regardless of content type.

Users’ social behaviour, measured in terms of volumes of activities, is not uniformly distributed: instead, it exhibits a heavy-tail distribution, where a minority of individuals is responsible for the great majority of content, as shown in figure 1. Furthermore, this result is confirmed even after stratifying for the type of content: in fact, a minority of users is actively involved in the production of false or misleading content in the context of COVID-19, contributing in a major way to the overall volume of fake news and to the possible over-representation of ideas that would otherwise be minor. Other users, instead, are characterized by a ‘bot-like’ behaviour, i.e. the tendency to passively retweet something without commenting on it or appending any original content, contributing nonetheless to its diffusion. Users within the platform may assume such misleading information as being representative of what the majority of users believes, which is the basis on which misperceived social norms can naturally emerge. Although these two qualitatively different types of users are both ‘fake news spreaders’—which is the term that we will use to identify them throughout this work—it is safe to assume that they will have different roles in the misinformation ecosystem. Users belonging to the first, active, group are highly motivated users, prepared to spend energy and time to craft messages (i.e. tweets) to spread them further. We will refer to users in this first group as ‘creators’. The second, passive, group contains those users who are more likely to engage in low-cost behaviours, such as retweeting. We will refer to users in this group as ‘consumers’. The latter are the ones that we suggest may be vulnerable to develop a misperception about the norm in place, namely acting in accordance with the perceived majority, even if he/she privately disapprove of the behaviour. Finally, outside of these two groups remains the rest of the online population, consisting of those user accounts that have never shared unreliable content but that, nonetheless, interact with creator and consumer through other kinds of retweets.

**Figure 2.**
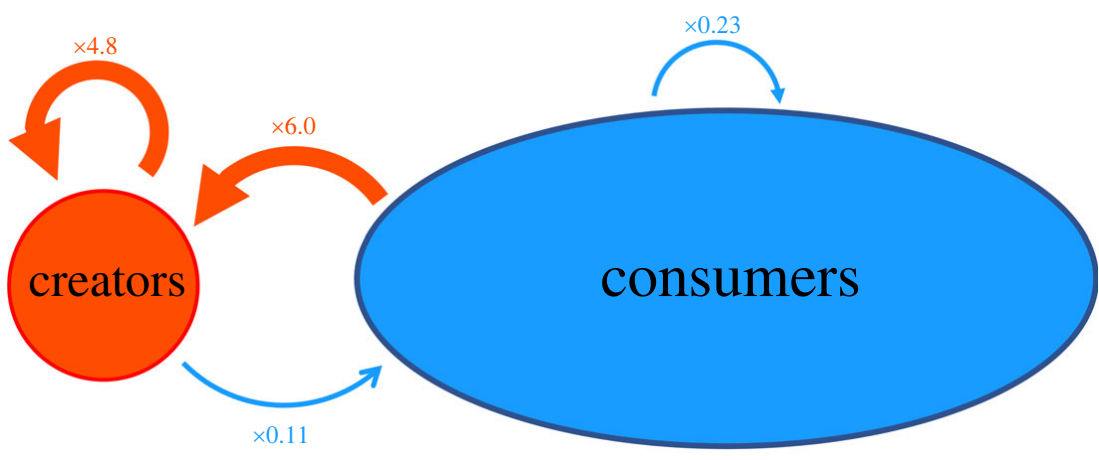
Schematic illustration of the separation between creators and consumers. The arrows represent the endorsements (i.e. retweets in Twitter) and go from the retweeting to the retweeted individuals. Values indicate the ratio between the observed number of links and the number one would expect if the links were randomly assigned, i.e. the number of links between groups were compared with that of an Erdös–Rényi model with the same number of total links. Note that the illustration is not at scale with numbers.

In order to talk more quantitatively about these two groups, it is useful to define them in terms of the fraction of fake retweets per user in a given time window. Specifically, we defined as creators all those users whose retweeted content in a given time interval is at least 20% fake. The creators’ group defined in this way contains 14% of the total users and it is responsible for 11% of retweets and the origin of 82% of fake content. On the other hand, the consumers’ group contains 86% of the users and is responsible for 89% of retweets and the origin of 18% of fake content. For more details about this choice see Methods and the electronic supplementary material, where an analysis of the difference in the number of followers between creators and consumers further supports our intuition behind the definition of the two groups (see figure 2).

**Figure 3.**
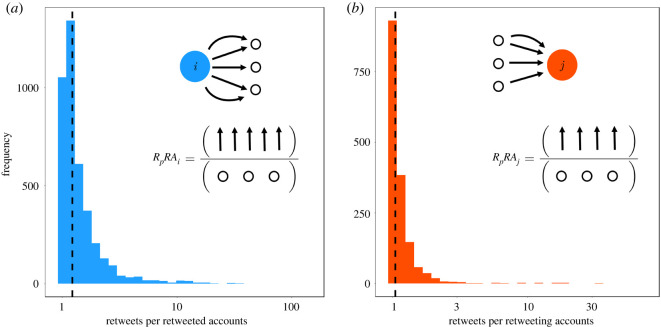
Do users engage always with the same accounts? To answer to this question, we computed the ratio of retweets per retweeted (retweeting) accounts in the case of consumers (creators), which are shown in (*a*,*b*), respectively. The acronym *RpRA* stands for what appears on the *x*-axis of each figure. In both cases, the distribution of this ratio among users is heavily skewed towards one, as shown by the black dashed line representing the median (1.2 and 1.04, respectively). This means that the majority of the users interacts with different people every time they retweet or are retweeted, therefore confirming the idea that on average people cannot understand what group (creators or consumers) they are interacting with. In the analysis users involved in less than 10 retweets are excluded because they would have further skewed the distribution without adding significant information.

Furthermore, to justify the claim that the separation of creators and consumers can act as a structural basis for the emergence of an ‘illusion of the majority’ we explore what may limit users’ opportunity to recognize the group belonging of the retweeted accounts (e.g. whether they are part of a minority or a majority). To do this, we computed and plotted the ratio of retweets per retweeted accounts (*RpRA*). If the users retweeted always the same accounts they could keep track and correctly infer the retweeted accounts’ group belonging. However our findings (figure 3) refute this possibility, showing that the median of the distribution of retweets per retweeted accounts (or, similarly, of retweets per retweeting accounts) is close to 1, meaning that the majority of users retweet each account only a single time. This suggests that the retweeting users have a limited understanding of the origin of the information they are sharing, which in turn makes it possible for a misperception about majority to arise.

### 2.2. Fluid transitions between creator and consumer groups

One of the consequences of defining both creators and consumers in terms of user activity is that when the underlying behaviour changes, the composition of the corresponding groups changes accordingly. Operationally, this dynamical behaviour over time leads to users going from being active spreaders to being silent, or vice versa, as well as anything in between. In practice, the groups of creators and consumers are fluid constructs that exist at every time step but are also continuously mutating and experiencing inward and outward user flows.

It is natural to wonder if the classification provided in the previous section—that of creators and consumers of fake news—still holds when such concepts are defined taking short time steps, e.g. 1 day, while allowing users to switch between groups. We find that only a small minority—specifically, 4.67%—of all fake news spreaders go from being creators to being consumers or vice versa. Moreover the majority of fake news spreaders tends to keep a similar spreading behaviour, even after a long period of inactivity (table 1).

**Table 1.** User behaviour in spreading fake news. Number of users classified according to their behaviour towards the spread of unreliable content: the majority of them tends to spread fake news only once, and, consequently, they were not taken into account in the analysis shown in figure 4. Also note that the majority of people becomes fake news spreaders only every once in a while. For instance, the users who go in and out of these groups less than 10 times are 96% of the total.

behaviour	users	only once	only twice	less than 10 times
only creators	13 848 (12.91%)	9804 (9.14%)	2027 (1.89%)	13 553 (12.64%)
only consumers	88 400 (82.42%)	58 328 (54.38%)	12 214 (11.38%)	85 967 (80.15%)
mixed	5005 (4.67%)	/	952 (0.88%)	3608 (3.36%)

**Figure 4.**
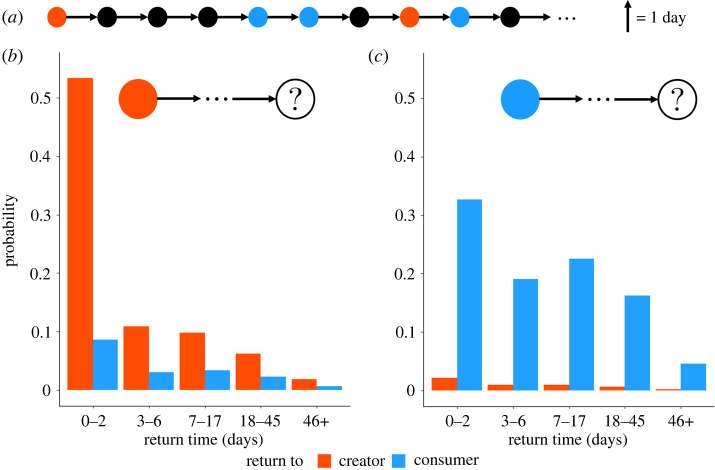
Fluid transitions between creators’ and consumers’ groups. Let us consider first-return times: (*a*) schematic example of the behaviour of a user (the circle), who might change his/her group at every time step (e.g. 1 day). Red, blue and black circles represent creators, consumers and non-spreaders, respectively. The return times are the number of black circles that separate coloured circles from one another, so in this example, they are 3, 0, 1 and 0 days, in chronological order from left- to right-hand side. The two histograms display the probability of returning, after a certain time, to a fake news spreading group (creators in orange, consumers in blue) for users that just stopped being (*b*) creators or (*c*) consumers.

Figure 4 shows how a user belonging to the creators’ (or consumers’) group is more likely to behave next, and after how much time. The analysis is restricted only to those users for which such a time can be unambiguously identified, meaning that we only consider those users that return to one of the two fake news groups within the time span of our temporal data. Our analysis shows two facts: first, the probability of returning to either one of the two fake news groups decreases with time; second, it is always more likely for users to return to the group that they left than to the other one. In practice, this means that creators are more likely to go back to being creators, no matter how much time they spent being silent, and the same holds for consumers. While these features are the same whether we consider creators or consumers, these two groups also display some notable differences. For instance users leaving the creators’ group are more likely to come back to it within 0–2 days, although in the same time span they can change group with 10% probability. Conversely, if a user starts from the consumers’ group they are much more likely to go back to it even after a long time, while the probability to change group in the same period is much lower, going down to 24 times less likely in the 18–45 day range. This makes this group more stable in terms of user dynamics and, consequently, more suitable to be considered for potential interventions. This finding supports our interpretation of consumers as those users who are not very committed to fake news spreading, since the time between consecutive fake news sharing is longer, on average, than that measured for the creators.

### 2.3. Unravelling causality between creators’ dynamics and fake news volume

**Figure 5.**
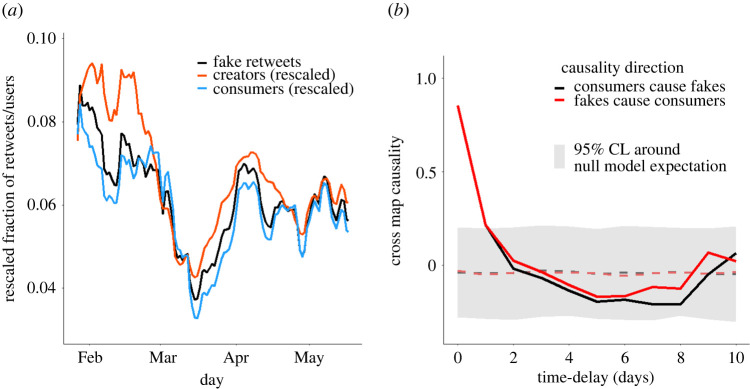
Unravelling causal relationships between group dynamics and fake news volume. (*a*) Comparison between the time series of the fraction of fake retweets (black line), the fraction of consumers (blue line) and the fraction of creators (red line). The latter were rescaled to ease the comparison between trends. The time step is of 1 day, while the lines are obtained through a 10 days moving average. (*b*) Cross-map signal computed for different time-delays with the convergent cross-mapping algorithm (see Methods). For the null hypothesis, we used surrogates obtained by randomly reshuffling empirical observations. The null hypothesis is rejected at 95% confidence level (CL), equivalent to an *a priori* test size of 5%, only at time delay equal to 0 and 1 day, with the strongest signal at the former.

It is plausible to ask whether the overall volume of fake news increases in response to an increase in the number of fake news spreaders or if it is the other way around. To inspect the existence of a causal relation between the size of the two communities of fake news spreaders and the overall volume of fake news circulating in the network, we consider temporal snapshots of 1 day for the analysis. For each time slice, we count how many creators and consumers there are and how many fake news items are being shared, building the three time series shown in figure 5*a*, which appear to be at least correlated with each other. First, we quantify such correlations: The cross-correlation between the fraction of creators and the fraction of fake content is 0.7; this value goes to 0.69 when we consider the fraction of creators and consumers and reaches 0.97 when we consider the last pair of time series, i.e. fraction of consumers and fraction of fake content.

Moving beyond simple correlations, we identify what is the cause–effect relation between the number of consumers and fake news spreading by means of the convergent cross-mapping (CCM) algorithm (see Methods). To investigate all the possible cases, we look for both short-time and long-time effects by varying the time-delay parameter that the method allows one to tune. Our results are shown in figure 5*b*, where the null hypothesis is obtained by performing the same causal test over different surrogates of our original time series, obtained by randomly permuting empirical observations while destroying any temporal correlation.

As shown in figure 5*b*, the cross-map causality, i.e. the quantity that indicates how strongly two time series are causally linked, is highest when the time delay is 0 days, and above the 95% confidence level (CL) only for 0 and 1 days for both the causality directions (consumers cause a higher volume of fake news and such a volume, in turn, cause a growth in consumers size). An analogous result is obtained when, instead of the consumers’ dynamics, creators’ dynamics is considered. Although a causal relation between the time course of the two empirical dynamics has been found, it is not possible to conclude which of the two quantities is responsible for the variation of the other, either because (i) there is a very strong feedback loop that propagates causal effects over fast time scales or (ii) the two variables are driven by a third, external or hidden, common cause. The proportionality between fake news and retweeters has already been shown in a study by González-Bailón *et al.* [47], but our analysis shows that the same result holds even if we only consider consumers and not all the accounts spreading fake news. These results provide additional support to the fact that due to their size the consumers are mainly the ones responsible for shaping the fake news ecosystem.

## 3. Discussion

Overall, from our analysis, a consistent picture arises. First of all, we were able to separate fake news spreaders into two groups: a small one (14%) of active and motivated users (creators) and larger one (86%) of users who instead prefer to repeat what other people say without creating content on their own (consumers). We did so by putting together information coming from the structural network such as the in-degree of each node with the information coming from the metadata on the retweets' nature, namely the subject of the message. Furthermore, the criterion that we chose proved to be robust against the variation of the only arbitrary parameter that it takes as input.

As a result, we were able to classify all users in the network based on their group’s belonging. These results are consistent with previous findings and provide additional support to the idea that on Twitter the communication network is characterized by a low degree of pluralism, since the majority of content circulating online is mainly produced by a minority of committed users [47–51,53]. Adopting a social norm perspective allows us to look at these findings through a new lens and provide novel insights on the social factors that could favour misinformation spread. Indeed, we suggest that the mismatch between the numbers of users producing content and of those that share it creates the structural bases on which specific social biases can arise. Specifically, the creator–consumer classification allows us to quantify their role in the spreading of fake news and to understand how much these types are susceptible to develop a wrong representation of the opinions of their community members. On the one hand, given that creators strongly interact among themselves, it is reasonable to think that they have a correct estimate of the opinions of the other members of their group. On the other hand, the consumers are users who read and retweet content coming from the creators. Since creators are only a minor fraction of the Twitter landscape, such an overexposure to their tweets may lead consumers to believe that the opinions of the creators are representative of those held by the majority of users (figure 3). This may generate a pressure to act accordingly to the (mis)perceived majority, even if this is not in line with their own private opinions. One may ask if such interpretation is specific to fake news or, alternatively, if it can apply also to other types of news (e.g. mainstream media, science) shared on social media. While such a creator–consumer pattern seems to occur regardless of the content expressed (e.g. figure 1), the effect in generating a misperception about the opinion of the majority is different when disinformation is shared. Specifically, it is unlikely that observing few users sharing reliable news would produce a wrong representation of the opinions of many, given that for the reliable content, what few do is already coherent with what most approve of, and is thus not expected to produce changes in the (already high) perceived social support of such behaviour. Instead, with regard to disinformation, what few do is probably not in line with what the majority considers the appropriate way to act, and it is exactly through such misalignment that misperception about the opinions of many can emerge.

One of the benefits of our distinction between creators and consumers is the possibility of doing a temporal analysis and therefore to judge how user behaviour and fake news circulation relate to each other. In our analysis, we found that it is impossible (at least with the time resolution available to us) to understand if fake news causes a growth in the number of highly active fake news spreaders or vice versa or, again, if they simply are the effect of a third, external event. Our results show that the number of consumers is the most strongly correlated with the fake news volume, since these two quantities show a stunning cross-correlation of 0.97. This confirms the idea that it is on consumers, those more susceptible to a possible ‘illusion of majority’, that one should focus in order to control and, hopefully, reduce the incidence of fake content online. We acknowledge the limits of the exploratory nature of this work. Specifically, future experimental work eliciting users private (and perception of others’) opinions, their actual diffusion, and the potential mismatch with what users share online is needed to confirm the causal effect of the aforementioned misperceptions on users’ online behaviour. As a precondition, it is, however, essential to explore if the naturally occurring structural features of social media communication could support the emergence of such social phenomena. Our findings make us confident that interpreting misinformation also through the lens of social norms can advance our understanding of the mechanisms supporting the spread of fake news and spark scientific discussions on how to improve the design of solutions to curb this phenomenon. For example, in offline contexts the effect of beliefs and opinions misperceptions can be alleviated by informing people about the real distribution of beliefs and expectations of others [54]. If, as anticipated by our work, such biases occur in online communication networks too, similar kinds of ‘bottom-up’ interventions could be designed to prevent users from sharing unreliable content in online settings. Specifically, unveiling the collective misunderstanding on which users' behaviour is based may reduce the pressure to act as the perceived majority does, thus allowing users to behave according to their (potentially disagreeing) private preferences. Such interventions may encounter less resistance and lead to less backfiring effects (e.g. increasing polarization and resistance to opinion change) compared with debunking information provided by external authorities or fact-checkers [55,56]. Our study makes several important contributions to the current literature addressing the expression of opinions in social media. We provide a novel perspective regarding the social mechanisms that may drive individuals to share unreliable content in online settings, complementing a growing body of interdisciplinary work exploring the social motives and the psychology that underpins the dynamics of social media sharing [57,58]. Misinformation spreading can be particularly dangerous in the context of the current COVID-19 pandemic as it may decrease people's willingness to comply with preventive behaviours (e.g. taking vaccines, wearing masks and adopting physical distancing) [14]. Although more evidence is needed to assess that online undesirable behaviour may spill over in offline behaviour (see also [59]), shedding light on the role of social factors—i.e. social norms—in the context of fake news spread may offer a novel approach for fighting misinformation and it is an attempt to respond to the current call from the research community for a better integration of the social sciences to support COVID-19 pandemic response [14].

## 4. Methods

### 4.1. Dataset origin and description

The datasets that we used in this work come from the COVID-19 Infodemics Observatory [16,52]. Tweets associated with the COVID-19 pandemics (coronavirus, ncov, #Wuhan, covid19, COVID-19, SARSCoV2, COVID) have been automatically collected using the Twitter Filter API.

The fraction of tweets included in our filter is limited by Twitter to 1% of the total, which instead provided us with a random subsample of all interactions. Nevertheless, recall of approximately 40% of all tweets associated with Coronavirus is estimated during these months. To reconstruct the communication network from the messages, we use the type of public pairwise interactions known as retweets.

In order to identify misinformative content, we used a database of web domains we constructed joining together multiple publicly available data sources [16]. This allowed us to classify the URLs, that are included in about 20% of tweets, as one of seven categories: science, mainstream media, satire, clickbait, political, fake/hoax, conspiracy/junk science. A fraction of about 14% of URLs were successfully classified in this sense (about 25% in the case of Italy). In this analysis, we considered as ‘fake’ messages associated with domains marked as clickbait, fake/hoax, conspiracy/junk science. We also restricted our analysis to those users classified as real people, and not bots, using the machine learning methodology described in [26].

Lastly, our analysis is focused on three countries: the USA, Italy and the UK (the last two only appear in the electronic supplementary material). To select accounts associated with a particular country, our data infrastructure are based on the geo-coding of the accounts’ textual self-declared location, which we are able to successfully map to a country of the world in 50% of cases. Finally, in the case of the USA and the UK, the data spanned from 22 January to 22 May 2020; for Italy, the period was instead between 22 January and 2 December 2020.

### 4.2. Definition of creator and consumer

The definition of the concept of creators and consumers is based on a criterion applied to each user individually. This criterion is based on the fraction of fake news produced: if among all the tweets that an account has produced 20% or more is fake, then that user is considered to be a creator. On the other hand, if this fraction is between 0% and 20%, the account is considered to be a consumer.

**Figure 6.**
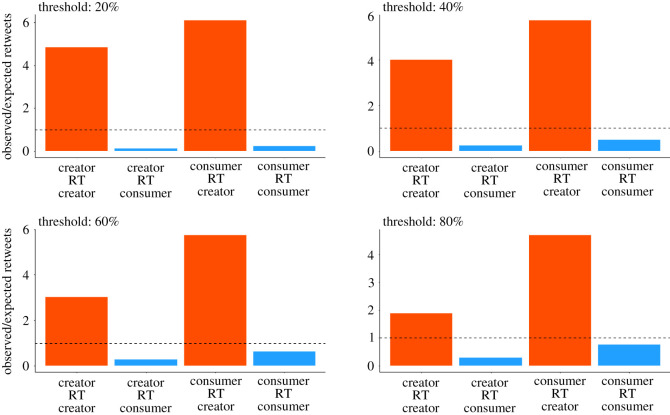
Analysis of the separation between creators and consumers for different definitions of these groups. The height of the bars indicates the ratio between the observed number of links between two groups and the number we would expect if the links were randomly distributed among the network (as in an Erdös–Rényi network). The dashed horizontal line corresponds to the case where the number of observed links is compatible with those of a random network (*y* = 1). The red and blue colours indicate if the tweet was originally from the creators’ or from the consumers’ group, respectively. The figures differ because of the threshold in the percentage of most active fake news spreaders used to define creators and consumers. However, it can be seen that the densities of the connections between these groups do not depend strongly on such a threshold.

In order to verify that this method is actually effective in identifying two separate groups, we compared our network against a null model with randomly distributed links (figure 6). In this way, we were able to highlight a clear separation between creators and consumers given by the fact that the former group is much more likely to be retweeted than the latter. Furthermore, this methodology allows us to carry out a sensitivity analysis to investigate the dependence of the behaviour of these systems on the threshold to be considered a creator (that we fixed to 20%). The result of such analysis shows that no matter what threshold we choose, this criterion is effective in separating fake news spreaders responsible for producing a large part of fake news, and those responsible for retweeting them, as shown in figure 6. Finally, in the electronic supplementary material, we show that the creators–consumers structure is not unique to the data from the USA but can be found in the data from Italy and the UK as well.

### 4.3. Causality detection via convergent cross-mapping

The algorithm that we use to infer the causal relation between the two time series in figure 5 is called convergent cross-mapping. It is based on a result known as Taken’s theorem, which allows us to reconstruct a dynamical system from the time series of a single one of its variables. The main idea behind this algorithm is that if we have two time series *x*_*t*_ (in our case the fake news volume) and *y*_*t*_ (the number of consumers), we can reconstruct for each of them a dynamical system which in turn gives a prediction of the original data, *M*_*x*_ in the case of *x*(*t*) and *M*_*y*_ in the case of *y*(*t*). Finally, in order to establish the causality relation between the two quantities we compute the Pearson coefficient between *x*(*t*) and the version of itself reconstructed from *M*_*y*_; the same is done with *y*(*t*) and *M*_*x*_. The only parameter we need to specify is the embedding dimension *E*, that we put equal to 10. Finally to generate the confidence interval, that is the grey area in figure 5, we repeat the same algorithm on 500 surrogates generated by randomizing the order of our time series.

For a more in in-depth discussion on the exact algorithm and the theory behind it see [60].

## Data Availability

Complying with the developer licence granted by Twitter, only the tweets identifiers can be made available. They can be found in the Dryad Digital Repository: https://doi.org/10.5061/dryad.bzkh189cc [61]. The code that we used for analysing the data can be found at https://github.com/PGcastioni/Creators_and_Consumers. The data are provided in electronic supplementary material [62].
